# Platelet inhibitory effects of the NO independent sGC stimulator riociguat (Bay 63-2561)

**DOI:** 10.1186/2050-6511-14-S1-P55

**Published:** 2013-08-29

**Authors:** Cora Reiss, Igor Mindukshev, Verena Bischoff, Stepan Gambaryan, Ulrich Walter

**Affiliations:** 1 Center for Thrombosis and Hemostasis, Johannes Gutenberg University Medical Center Mainz, Mainz, Germany; 2 Sechenov Institute of Evolutionary Physiology and Biochemistry, Russian Academy of Sciences, St. Petersburg, Russia; 3 Institute of Clinical Biochemistry and Pathobiochemistry, University of Würzburg, Würzburg, Germany

## Background

Nitric oxide (NO) is a potent biological mediator and plays an important role in cardiovascular homeostasis. Here it activates the soluble guanylyl cyclase (sGC) that synthesizes cGMP. The intracellular second messenger cGMP regulates vascular tone, vascular permeability, modulates inflammation and platelet reactivity. In platelets cGMP activates platelet inhibitory pathways via PKG and thereby inhibits platelet activation. Many cardiovascular diseases e.g. pulmonary arterial hypertension (PAH), are associated to a defective NO signalling. Therefore agents that stimulate the sGC are interesting therapeutic tools for the treatment of several cardiovascular diseases.

Riociguat (Bay 63-2561) stimulates the sGC NO-independently without the risk of nitrate tolerance. Clinical Phase II and III studies determined the efficacy and tolerability in pulmonary hypertension patients. Riociguat was well tolerated and also significantly improved exercise capacity and hemodynamic parameters. To further analyse the effects of riociguat on platelet activation we tested the inhibitory effects on functions in isolated platelets and whole blood to elucidate additional applications of riociguat and to estimate adverse effects such as increased bleeding tendencies.

## Results

In order to study the effects of riociguat on platelet sGC activity we monitored the VASP phosphorylation and cyclic nucleotides in washed platelets. Already low therapeutically relevant doses of 0.1 µM riociguat increased cGMP levels and VASP phosphorylation significantly. Consistent to these findings ADP induced platelet activation in washed platelets, as shown by platelet shape change, aggregation and integrin activation, was significantly impaired using low doses of riociguat. Additionally adhesion on collagen under shear conditions is significantly reduced in platelet riched plasma. In contrast to that one hundred times higher concentrations of riociguat were needed to obtain a significant increase of VASP phosphorylation as well as a significant inhibition of ADP induced aggregation in whole blood.

## Conclusion

Our results indicate that riociguat treatment will not lead to an increased bleeding tendency in pulmonary hypertension patients. Additional possible applications of riociguat, where targeting platelet function is beneficial, might be achieved using higher doses of riociguat.

